# eRegTime, Efficiency of Health Information Management Using an Electronic Registry for Maternal and Child Health: Protocol for a Time-Motion Study in a Cluster Randomized Trial

**DOI:** 10.2196/13653

**Published:** 2019-08-07

**Authors:** Marie Hella Lindberg, Mahima Venkateswaran, Khadija Abu Khader, Tamara Awwad, Buthaina Ghanem, Taghreed Hijaz, Kjersti Mørkrid, J Frederik Frøen

**Affiliations:** 1 Faculty of Health Sciences UiT – the Arctic University of Norway Tromsø Norway; 2 Global Health Cluster Division for Health Services Norwegian Institute of Public Health Oslo Norway; 3 Centre for Intervention Science in Maternal and Child Health University of Bergen Bergen Norway; 4 Palestinian National Institute of Public Health World Health Organization Al-Bireh Occupied Palestinian Territory; 5 Ministry of Health Ramallah Occupied Palestinian Territory

**Keywords:** health information systems, eHealth, Time and Motion Studies, workflow, antenatal care, developing countries

## Abstract

**Background:**

Paper-based routine health information systems often require repetitive data entry. In the West Bank, the primary health care system for maternal and child health was entirely paper-based, with care providers spending considerable amounts of time maintaining multiple files and client registers. As part of the phased national implementation of an electronic health information system, some of the primary health care clinics are now using an electronic registry (eRegistry) for maternal and child health. The eRegistry consists of client-level data entered by care providers at the point-of-care and supports several digital health interventions that are triggered by the documented clinical data, including guideline-based clinical decision support and automated public health reports.

**Objective:**

The aim of the eRegTime study is to investigate whether the use of the eRegistry leads to changes in time-efficiency in health information management by the care providers, compared with the paper-based systems.

**Methods:**

This is a substudy in a cluster randomized controlled trial (the eRegQual study) and uses the time-motion observational study design. The primary outcome is the time spent on health information management for antenatal care, informed and defined by workflow mapping in the clinics. We performed sample size estimations to enable the detection of a 25% change in time-efficiency with a 90% power using an intracluster correlation coefficient of 0.1 and an alpha of .05. We observed care providers for full workdays in 24 randomly selected primary health care clinics—12 using the eRegistry and 12 still using paper. Linear mixed effects models will be used to compare the time spent on health information management per client per care provider.

**Results:**

Although the objective of the eRegQual study is to assess the effectiveness of the eRegistry in improving quality of antenatal care, the results of the eRegTime study will contribute to process evaluation, supplementing the findings of the larger trial.

**Conclusions:**

Electronic health tools are expected to reduce workload for the care providers and thus improve efficiency of clinical work. To achieve these benefits, the implementation of such systems requires both integration with existing workflows and the creation of new workflows. Studies assessing the time-efficiency of electronic health information systems can inform policy decisions for implementations in resource-limited low- and middle-income settings.

**International Registered Report Identifier (IRRID):**

DERR1-10.2196/13653

## Introduction

### Background

Robust health information systems play a central role in the strengthening of health systems and achieving universal health coverage [1-4]. There are, however, substantial gaps in the reliability, timeliness, and efficiency of health data collection, analysis, and use in many countries, hampering evidence-based decision making at all levels of the health system [5]. Common traits of many health systems include inefficient and uncoordinated data processing and management [6]. Health care providers are often obliged to repeatedly collect, compile, and report redundant health information. Therefore, time spent on direct patient care might be shortened [5,7]. The introduction of health information technologies could substantially influence care providers’ workflow and clinical work processes [8,9]. Existing evidence, primarily from high-income contexts, suggests that access to relevant health information tends to improve with the use of electronic health information systems but is often associated with time-consuming and counterintuitive user-system interactions [10-14]. There is limited evidence from low- and middle-income countries (LMICs) on how the use of electronic health information systems affect clinical workflow or efficiency [15,16]. LMIC can least afford wasting the time of a limited health workforce and may gain the most from improved efficiency of health information management [17,18]. It is therefore important to detect and understand the specific challenges faced in settings with fewer resources to successfully and sustainably implement electronic health information systems in such contexts.

In the West Bank, Palestine, an electronic health information system—the eRegistry for maternal and child health—is currently being rolled out on a national scale throughout primary health care. The eRegistry consists of electronic health (eHealth) records for antenatal, postpartum, and newborn care for use at the point of care by the care providers. The eRegistry supports automated clinical decision support, workflow management support, and referral functionalities. [1,19-21]. Care providers in primary health care clinics can access the eRegistry through desktop computers where they enter all client-related information [22]. The Palestinian eRegistry is installed in the District Health Information System 2 (DHIS2) tracker software; DHIS2 is a Web-based platform that is free and open-source and currently in use in more than 50 low- and middle-income settings largely for collection of aggregate data in a health information system or, to a lesser extent, for individual-level data in the health system.

An ongoing cluster randomized controlled trial (CRCT), the eRegQual study, is embedded in the national implementation of the eRegistry, where clinics using the eRegistry were included in the intervention arm and compared with the control arm that used paper-based records [22]. The primary objective of the eRegQual CRCT is to assess the effectiveness of the eRegistry in improving health outcomes for pregnant women and process outcomes of quality of antenatal care. Further details of the eRegQual study can be found in the published trial protocol [22].

The time-motion method is one of the more robust study designs for the collection and quantification of time data [8] and has been used to study costs and inefficiencies in the delivery of health care as well as patient safety and quality [23]. The time-motion study design in health care involves continuous observations of clinicians’ work in health facilities by recording the time taken to perform a set of predefined tasks. This study design is frequently applied in assessing whether the introduction of an eHealth tool is associated with changes in time-efficiency [24].

### Objectives

The aim of the eRegTime study is to evaluate whether the use of an eRegistry changes the time-efficiency of care providers in primary health care clinics for antenatal care. Time-efficiency will be assessed by measuring the time spent by the care providers on health information management.

## Methods

In this protocol, we have followed the Suggested Time and Motion Procedures checklist for standardized reporting of studies using the time-motion design (see Multimedia Appendix 1) [8] as well as the Standard Protocol Items: Recommendations for Intervention Trials checklist (see Multimedia Appendix 2).

### Setting

In the West Bank, Palestine, primary health care clinics provide antenatal, postpartum, and newborn care.

The different cadres of health care providers that work in maternal and child health in primary health care clinics include midwives, nurses, general practitioners trained in maternal and child health care, and specialist obstetricians. Smaller clinics (less than 50 new enrollments of pregnancies a year) typically have a nurse or a midwife working throughout the week, whereas the doctor visits the clinic once every 2 weeks. Larger clinics (more than 50 new enrollments of pregnancies a year) and referral clinics have specialist obstetricians, in addition. The nurse or midwife in the clinics does the majority of the antenatal care consultation and after-consultation work that involves health information management and were the only groups of care providers observed in this study.

The phased national implementation of the eRegistry was undertaken in tandem with the eRegQual study; the intervention clusters of the eRegQual study were the clinics that received the eRegistry as part of phase 1 of implementation, whereas control clusters continued to use paper-based clinical records and were scheduled to receive the eRegistry after the end of the eRegQual study. Although 68 primary health care clinics started using the eRegistry in phase 1 of implementation, and 59 clinics continued to use paper-based clinical records, some of the smaller clinics were clubbed to form clusters before randomization for the eRegQual study. Larger clinics were considered as clusters of their own. In total, there were 60 clusters in each arm of the eRegQual study. Details of enrollment and randomization for the eRegQual study can be found in the published trial protocol [22].

The intervention evaluated in the eRegQual study—the eRegistry—is used as a point-of-care electronic data entry tool in primary health care clinics in the West Bank [22]. Guideline-based clinical decision support and automated electronic monthly reports are 2 digital health interventions currently supported by the Palestinian eRegistry. The eRegistry is intended to fully replace paper-based systems for maternal and child health in primary health care in the West Bank.

### Workflow in Primary Health Care Clinics

#### Workflow in Clinics Using Paper-Based Systems

Pregnant women visit primary health care clinics for their first antenatal (booking) visit on specific workdays (clinics may work 1-4 days a week). The nurse or midwife in the clinics receives the pregnant women for the booking visit and documents a set of demographic data (eg, name, national identification number, address, phone number, and date of birth), and medical, surgical, and obstetric history. Afterward, the nurse or midwife measures and documents the woman’s height and weight, blood pressure, and fundal height and orders and fills out routine laboratory results appropriate for each antenatal visit. As part of the booking visit, the doctor examines women on the same workday or a few workdays later in some clinics. The nurse or midwife assists the doctor in medical and ultrasound examinations. For pregnant women identified with risk factors that warrant a referral, the nurse or midwife makes necessary arrangements for transfer to the referral health facility. There is a flexible appointment system for all subsequent antenatal visits. For uncomplicated pregnancies, the nurse or midwife documents blood pressure and fundal height, checks for fetal presentation, and orders laboratory investigations during the subsequent antenatal visits. Nurses and midwives typically do client care for pregnant and postpartum women as well as newborns in the first part of the workday. Following this, the nurse or midwife usually completes registers for antenatal care, referrals, ultrasounds, vaccines, and laboratory investigations. The nurse or midwife also compiles the data in the registers for public health reporting to the Palestinian Ministry of Health, typically concentrating this task in 1 or 2 workdays monthly. Event counts of number of pregnancies registered in the clinic, number of ultrasound examinations and laboratory tests that are performed, and number of pregnancies with risk conditions that are referred are some examples of the data that are part of standardized monthly reports submitted by care providers [25].

#### Workflow in Clinics Using the Electronic Registry

All clinical tasks, as described for the control clusters, are identical in case of the intervention clusters. Only the health information management differs. The eRegistry is used by care providers to document real-time clinical data during client consultation. On the basis of the data entered at the point of care, the eRegistry generates automated decision support and workflow assistance [19,22]. Laboratory systems are not integrated in the eRegistry, and care providers need to enter the laboratory results they receive on paper into the eRegistry retrospectively. The eRegistry aggregates and submits all data that are part of the public health reports automatically every month to the Palestinian Ministry of Health.

### Study Design

The time-motion study design was employed to collect data in the eRegTime study [8,24]. Observations were conducted in a randomly selected subsample of intervention and control clusters (primary health care clinics) of the eRegQual CRCT.

#### Outcome Measures

The primary outcome measure is the time spent on health information management per consultation. We defined health information management as the preparations and executions of collection, aggregation, analysis, and dissemination of clinical data, both at the individual and aggregate levels [26]. To tailor the general definition of the primary outcome to fit our context, we first used workflow mapping exercises ahead of data collections for the eRegTime study (as described previously) to list all the tasks usually done by the nurse or midwife in the primary health care clinics during antenatal care on a typical workday [27]. We then defined 6 activity types corresponding to the tasks: accessing information, reporting, documentation, client care, client-related care, and miscellaneous. The primary outcome measure—health information management time—was defined as time spent on all tasks involving the activity types “information access,” “information documentation,” and “information reporting” (see Table 1) [27]. “Information access” includes all activities that involve seeking and finding relevant existing health or demographic information on the client [27]. “Information documentation” consists of all tasks that involve writing down client information in the antenatal records (electronic or paper), laboratory, and ultrasound forms [27]. “Information reporting” is defined as transferring information from the antenatal records and registers for public health reporting [27].

A total of 2 additional analysis categories were defined: (1) “client care” that includes all activities in which the care provider is fully focused on the client without any writing and (2) “client-related care” that refers to all tasks that are imperative for care of individual pregnant women undertaken between 2 antenatal care consultations (see Table 1).

Activities unrelated to care of clients, including personal activities of the care providers, and tidying and preparing the consultation room for new clients, were categorized as “miscellaneous” (see Table 1) [27].

**Table 1:** Analysis categories including the primary outcome measure, corresponding task, and task category as defined for data collection (adapted from the study by Pizziferri et al [30] and tailored to the local context).

Analysis category	Task category in data collection tool	Name of task in data collection tool
Client care	Outside	Assisting doctor
Client care	Outside	Examination in other room
Client care	Procedures	Clinical and medical examination
Client care	Procedures	Injections and bloodtake
Client care	Procedures	Giving tablets
Client care	Procedures	Other
Client care	Talking	Education and counseling
Client care	Talking	Talking to family
Client care	Talking	History: demographic and medical
Client care or client-related care^a^	Talking	Clinical support
Client care or client-related care^a^	Talking	Call client or family
Client care or client-related care^a^	Talking	Referrals
Client care or client-related care^a^	Talking	Other
Health information management	Between or after consultations	Writing in statistics book
Health information management	Computer-Find	Client file
Health information management	Computer-Find	Lab or ultrasound results
Health information management	Computer-Writing	Client file (including history)
Health information management	Computer-Writing	Lab or ultrasound form
Health information management	Computer-Writing	Schedule appointment
Health information management	Computer-Writing	Text message in eRegistry
Health information management	Paper-Find	Client file
Health information management	Paper-Find	Lab or ultrasound results
Health information management	Paper-Writing	MCH (Maternal and Child Health) Handbook (including history)
Health information management	Paper-Writing	Client file (including history)
Health information management	Paper-Writing	Register book
Health information management	Paper-Writing	MCH Handbook or register book
Health information management	Paper-Writing	Register book or client file
Health information management	Paper-Writing	Client file or MCH handbook
Health information management	Paper-Writing	Lab, ultrasound, prescriptions, and referrals
Health information management	Paper-Writing	Schedule appointment
Health information management	Paper-Writing	Writing on other paper
Health information management	Talking	Explaining test results
Health information management	Talking	Technical support
Health information management or client-related care^b^	Computer-Read	Appointment list
Health information management or client-related care^b^	Computer-Read	Client file
Health information management or client-related care^b^	Computer-Read	Lab or ultrasound results
Health information management or client-related care^b^	Computer-Read	Guidelines, treatment
Health information management or client-related care^b^	Computer-Read	Other info
Health information management or client-related care^b^	Paper-Read	Appointment list
Health information management or client-related care^b^	Paper-Read	MCH handbook
Health information management or client-related care^b^	Paper-Read	Client file
Health information management or client-related care^b^	Paper-Read	Lab or ultrasound results
Health information management or client-related care^b^	Paper-Read	Treatment guidelines
Health information management or client-related care^b^	Paper-Read	Other info
Miscellaneous	Between or after consultations	Cleaning, arranging files
Miscellaneous	Between or after consultations	Phone and computer (personal)
Miscellaneous	Between or after consultations	Other: praying, eating, toilet
Miscellaneous	Between or after consultations	Eating, praying, toilet
Miscellaneous	Between or after consultations	Group education
Miscellaneous	Postpartum care	Postpartum care

^a^Task classified as client-related care if done outside of a consultation. If done within an antenatal care consultation, it is classified as client care.

^b^Task classified as client-related care if done outside of a consultation. If done within an antenatal care consultation, it is classified as health information management.

#### Eligibility Criteria

**Figure 1:**
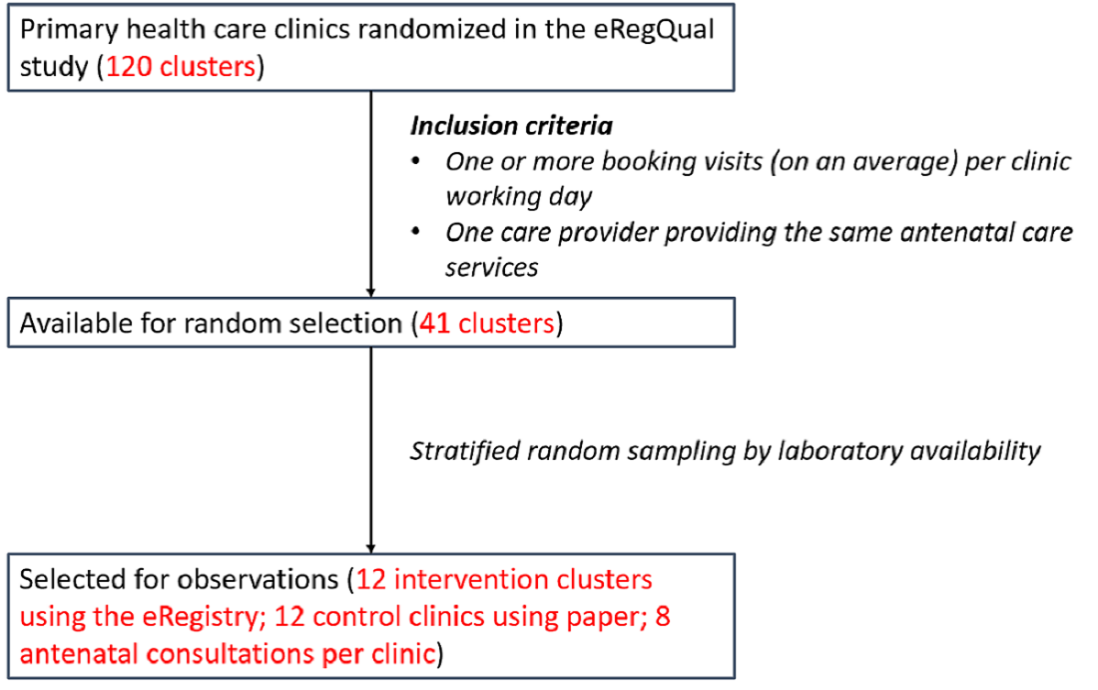
Selection of clusters in primary health care clinics for observations.

Clusters (primary health care clinics) that are part of the eRegQual CRCT that fulfil the following criteria were eligible for inclusion in the eRegTime study: (1) Have 1 nurse or 1 midwife providing antenatal care services on a given workday (to maintain a 1:1 subject-to-observer ratio) and (2) Have, on average, at least 1 booking visit per workday (to ensure capturing a sufficient number of antenatal booking visits). After applying these inclusion criteria to the 120 clusters that are part of the eRegQual CRCT, 41 clusters were eligible for the time-motion study observations (20 intervention clusters and 21 control clusters; Figure 1).

#### Sampling

For sample size estimations, we assumed that clinics using paper-based systems spend an average of 10 min on health information management per client. We also assumed unequal and higher SD around the mean health information management time (in minutes) for clinics that use the eRegistry (SD=5) compared with clinics that use paper-based systems (SD=2) because of an expected variance in computer literacy and confidence of use. Sample size calculations were made using the Stata command “clustersampsi” to detect a 25% difference at a 90% power and 5% significance using an a priori intracluster correlation coefficient of .1 [28,29]. A total of 24 primary health care clinics were selected to be observed, 12 from each arm of the CRCT, with at least 8 observed antenatal consultations per clinic (Figure 1). Statisticians that are independent of the eRegTime study team performed a random sampling of the primary health care clinics for the observations, stratified on laboratory availability.

#### Data Collection Methods

**Figure 2:**
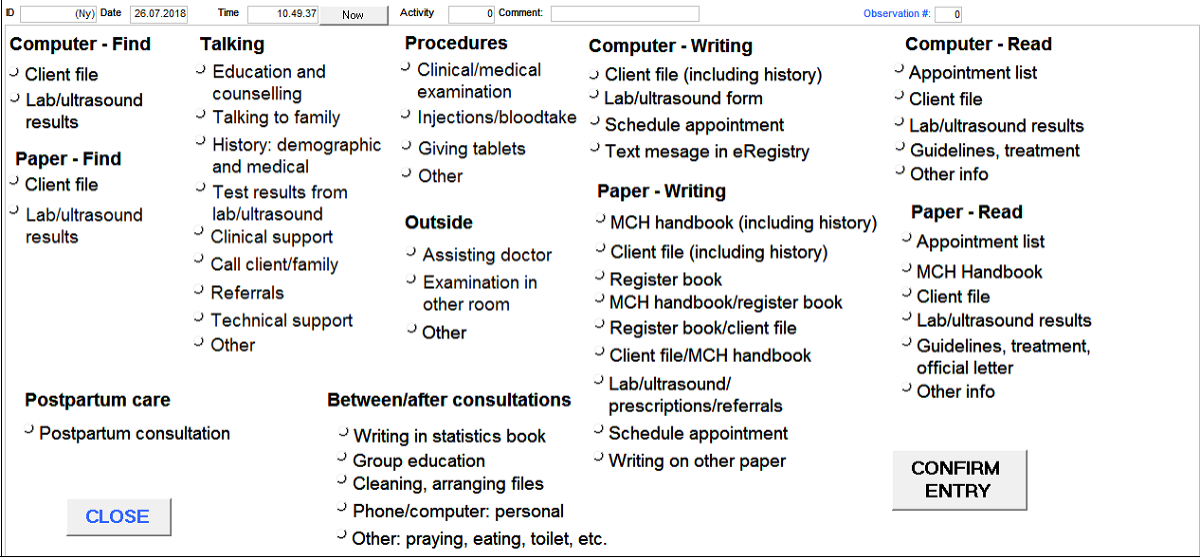
Data collection tool (data entry form) used for recording time-motion data.

We designed the data collection tool based on a Microsoft Access database template made available online by the US Agency for Healthcare Research and Quality [24], customized to the clinical workflow in the West Bank. The data collection tool was installed on handheld tablets. The data collection tool contains a list of tasks categorized under 10 task categories, and every task can be time-stamped (Figure 2) [30,31]. The task categories covered the care providers’ entire workday consisting of every clinical and nonclinical task, including after-consultation and between-consultation work.

The observers were trained to first determine the nature of the observed task and then click on the corresponding task on the data collection tool (Figure 2) [32]. The observer could end a task by clicking on the “confirm entry” button (Figure 2). In case of multitasking by the care providers, the observers were instructed to select the principal activity. After-consultation work were recorded as separate observations, as were postpartum care consultations.

The database stored the observation times for each task with an activity code linked to the tasks. In accordance with ethical approvals for the study, no personal or other demographic data related to the client or the care provider were collected, and clinic names were only being stored as computer-generated codes in the database.

A total of 4 trained observers completed the data collection. Observers were trained with simulation videos on the time-motion methodology and the task categories and in using the data collection tool (see Multimedia Appendix 3). Following training, the observers conducted practice observations in nonstudy clinics with and without the eRegistry. After this, observations and data collections were undertaken in the study clinics. The observers recorded a full workday and included all the antenatal consultations during that day. If the required number of antenatal consultations per clinic (n=8) was not achieved in 1 day, additional days of observation were carried out until the required cluster size was reached.

The field coordinators of the study received the data after each day of data collection and checked that the sample size for each clinic is reached with a sufficient number of documented observations.

#### Blinding

Although neither the observers nor the care providers in the primary health care clinics can be blinded to the intervention, they both will be blinded to the outcomes of the eRegTime study and have only been informed of the overarching objective of the eRegQual CRCT (including the eRegTime study) of assessing effects of the eRegistry on the quality of care. To ensure blinding of the observers to the outcome, the data collection tool included an exhaustive list of tasks, beyond the primary outcome of the eRegTime study (see Table 1; Figure 2).

### Data Analyses

The unit of measurement of the primary outcome is the time spent on health information management per client per care provider, where time will be analyzed in minutes. Statistical analyses will be performed using Stata version 15 or later (StataCorp LLC, 2017,  *Stata Statistical Software: Release 15*) or RStudio version 1.2.1335 or later. Descriptive statistics for the time variables will be summarized as means and SDs. We will report on the average time spent on antenatal consultation overall, and for booking visits and other antenatal visits separately, and the average time spent on each of the activity types including those that are not part of the primary outcome (including “client care,” “client-related care,” and “miscellaneous”; see Table 1).

We assume that the nurse or midwife spends, on average, an equal amount of time on after-consultation documentation work per client. The after-consultation time spent on client-related documentation and public health reporting will be averaged over the number of observed antenatal consultations and added to the time spent per consultation. Differences in the health information management time between the clinics with and without the eRegistry will be tested for significance using the linear mixed effects model to account for clustering [33,34]. In addition, as secondary analyses, we will test for differences in the health information management time separately for booking visits and other antenatal visits, and differences in time spent on other activity types in the 2 arms. Postpartum care consultations will be excluded from the analysis, as the focus of both eRegQual and eRegTime studies is on antenatal care quality and clinical processes.

Accompanying the results of the outcomes of the study, interobserver reliability assessments will be reported using kappa coefficients for the total number of clinical tasks and activity types recorded and intraclass correlation coefficients for the recorded mean times for the tasks and analysis categories (see Table 1) [35].

### Ethics Approval and Consent to Participate

The eRegTime study was approved by the Palestinian Health Research Council (PHRC/HC/208/17) and the Regional Committee for Medical and Health Research Ethics in Norway (2017/400). Permissions to conduct observations in the clinics have been obtained from the Palestinian Ministry of Health. Care providers and supervisors of the primary health care clinics will be informed of the data collection for this study. Considering the local sensitivity and hesitance related to signing documents in our study context, pregnant women will be asked for oral consent to allow the observers to be present in the rooms during consultations, and the ethics committees were notified of this. No data will be recorded on personal or individual characteristics of pregnant women, care providers, or primary health care clinics. Only completely anonymous data will be available to the researchers for analysis.

## Results

Ethical approvals for conduct of the study were obtained in April 2017. The data collection tool was designed, tested, and adjusted over 2017 and 2018, followed by which the sample was selected for the main study. Clinics included in the sample were informed about the study before start of observations, and the data collection for the eRegTime study was completed between August and December 2018. Data will be analyzed for outcomes in July and August 2019; the results are expected to be published in the second half of 2019.

## Discussion

The eRegTime study is one of the few studies that assesses the impact of an eHealth intervention on clinical workflow and time-efficiency in a middle-income context, where the impact of using digital tools routinely during clinical care is probably much bigger given the manpower and resource constraints than high-income settings.

Most studies that have assessed the time-efficiency using the time-motion design find no statistical differences in the workload of care providers following the introduction of eHealth tools [15,30,31,36]. Factors that may potentially affect the time-efficiency of care providers while using eHealth tools are duration of use of eHealth tools, computer literacy, multitasking, and interruptions [37-41]. In some settings, a period of 18 to 24 months between the implementation of the eHealth tools and the observations was considered sufficient for the stabilization of clinical work routines [15,42]. Primary health care clinics observed in the eRegTime study will be using the eRegistry for a median time of 20 months at the time of the observations. According to a questionnaire survey conducted in the intervention clusters of the eRegQual CRCT, a quarter of the nurses and the midwives had never used a computer before starting to use the eRegistry. Formative research and workflow mapping exercises showed that health care service delivery in the setting of this study was characterized by fragmented workflow and the time taken to perform the different tasks was relatively short [27]. The time-motion design is particularly suitable for data collection in such settings [30,31]. Although interruptions to the workflow and multitasking might be overlooked because of the fact that the data collection tool requires the observer to select only 1 activity at the time, the primary objective of the eRegQual study is to assess quantitative differences in time spent between clinics with and without the eRegistry; data collection methods were designed to be identical in the 2 arms.

Other methods such as work sampling and self-reported questionnaires were considered during the planning phase of this study. However, with work sampling, in which activities are recorded only at certain time intervals, there is a risk of missing certain activities. In addition, this method requires an enormously large sample size, which was not feasible in our setting [43]. The use of self-reported questionnaires poses risks of inaccurate reporting and recall bias as well as being a considerable interference to the care provider’s workflow [44]. The time-motion design was therefore considered the most suitable for this study.

We acknowledge that it may not be possible to completely eliminate the risk of care providers behaving differently because they know they are being observed [45]. We will attempt to minimize this effect by training the observers to avoid interfering with clinical work [45]. Another potential source of bias is diverging subjective interpretations of the tasks during data collection by the observers, and this will be minimized by hands-on training sessions, practice sessions with simulation videos, and “test” observations before the start of the study observations.

## Appendix

Multimedia Appendix 1 Suggested Time and Motion Procedures (STAMP) checklist.

Multimedia Appendix 2 SPIRIT protocol checklist.

Multimedia Appendix 3 Training manual.

## Abbreviations

CRCT: cluster randomized controlled trial
DHIS2: District Health Information System 2
eHealth: electronic health
eRegistry: electronic registry
LMIC: low- and middle-income country
