# Supplementary material for: eRegTime, Efficiency of Health Information Management Using an Electronic Registry for Maternal and Child Health: Protocol for a Time-Motion Study in a Cluster Randomized Trial
Source: JMIR Res Protoc. 2019 Aug 7;8(8):e13653. doi: 10.2196/13653 (PMC6702800; doi:10.2196/13653)
Supplement: Multimedia Appendix 1 [file resprot_v8i8e13653_app1.pdf]

Suggested Time and Motion Procedures (STAMP) checklist

Area and element		Description
Intervention	Type	Electronic registry for maternal and child health (eRegistry)
	System genre	DHIS2 Tracker eRegistry
	Maturity	June-October 2016
Empirical setting	Institution type	Public clinics reporting to the Ministry of Health
	Care area	Mid-size primary healthcare clinics offering antenatal care
	Locale	Semi-urban
Research design	Protocol	Observational study within a CRCT
	Duration	Eight to ten weeks
	Shift distribution	Antenatal care working days
	Observation hours	Approximately 130 hours
Task category	Definition and classification of outcomes	See Table 1
Observer	Size of field team	Four independent observers
	Training	Training conducted according to the training manual
	Background	Nursing and public health, maternal and child healthcare
	Inter-observer uniformity	Will be reported using kappa coefficients for the total number of clinical tasks recorded, and intra-class correlation coefficient for the recorded mean times for the activity types (table 2).
	Continuity	Not applicable
	Assignment	To be determined
Subject	Size	24 primary healthcare clinics, 12 in each arm, with eight pregnant women consultations per clinic
	Recruitment and randomization	Sub-sample from eRegQual cluster randomized controlled trial. After applying exclusion and inclusion criteria, 43 primary healthcare clinics available for inclusion.
	Continuity	Not applicable
	Background	Nurse-midwives
Data recording	Multi-tasking	Multitasking not captured
	Non-observed periods	The observers will be instructed to remain in the consultation room the entire workday. If the nurse-midwife leaves the room, the observer will determine what the purpose of leaving the room is, e.g. to assist the client to the doctor, or to get the client's file
	Between-task transition	The beginning of a consecutive task marks the ending of the previous one
	Collection tool	Microsoft Access on Windows tablets, adapted from the template made available by the Agency for Healthcare Research and Quality (2).
Data analysis	Definition of key measures	Primary outcome: mean time spent on health information management.<br>Unit of analysis: time in minutes per consultation per nurse-midwife.
	Analytical methods	Descriptive characteristics will be presented as the number of observations with the mean and standard deviation, or the median and interquartile range.

		Differences between groups will be tested for significance using the linear mixed effects model.
Ancillary data	Interruption	Not applicable
	Interaction	Seeking assistance from colleagues via oral communication
	Location	Low-risk pregnancies, mid-size primary healthcare clinics (50 – 150 new pregnancy enrolments); in consultation rooms for antenatal care.

References:

Pizziferri L, Kittler AF, Volk LA, Honour MM, Gupta S, Wang S, et al. Primary care physician time utilization before and after implementation of an electronic health record: A time-motion study. Journal of Biomedical Informatics. 2005;38(3):176-88.
