# Supplementary material for: eRegTime, Efficiency of Health Information Management Using an Electronic Registry for Maternal and Child Health: Protocol for a Time-Motion Study in a Cluster Randomized Trial
Source: JMIR Res Protoc. 2019 Aug 7;8(8):e13653. doi: 10.2196/13653 (PMC6702800; doi:10.2196/13653)
Supplement: Multimedia Appendix 3 [file resprot_v8i8e13653_app3.pdf]

Training manual for observers

Introduction

This study is a so-called time-motion study, in which we want to know how much time care providers spend on performing different activities, and compare the time spent in the clinics that use the eRegistry versus those who still use paper. The observers' role will be to take the time on all the various tasks that care providers do during an antenatal care workday in primary healthcare clinics. We have developed a data collection tool in the software Microsoft Access. The tool contains a list of activities, and the observer is supposed to click on the corresponding button according to the activity she observes. The time will then automatically be stored in a database linked to the data entry form, which then can be used for analysis. The tool template has been downloaded from the Agency for Healthcare Research and Quality's website: https://healthit.ahrq.gov/health-it-tools-and-resources/time-and-motion-studies-database (under "Resources for Time and Motion Studies"). It has further been adjusted to our setting.

The observers will use tablets with Microsoft Access installed on it. During the observations, the observers will sit on a chair in the consultation room. It is important that the observer is sitting in a place where she can clearly observe what the care provider is doing, while at the same time keeping a distance in order to avoid any interruption of the care provider's work.

Description of the tool

The tasks in the tool are sorted into major and minor task categories. The major ones reflect the physical action used to perform the task e.g. talking, writing on the computer, or reading on paper. The minor categories are the actual task performed, e.g. reading in the client's paper file. Combined, these constitute the total amount of tasks performed by the care providers. The major categories are depicted with a bold font (see Table 1 and Figure 1 below). Only one task can be captured at a time. If the care provider is doing multiple activities at the same time, the observer must determine by the nature of the task which one to record. For example, if the care provider is writing in the client file at the same time as she is taking the client's history, this will be recorded as "Paper – writing – file", and not "Talking – history-taking" since the care provider is *primarily* writing. Talking will therefore always come second when the care provider is talking at the same time as doing something else. History-taking is in this case included in "paper – writing – file", since the woman's history is written down in the file. See detailed description in the table below (Table 1).

Table 1: Detailed description of the tasks according to the major (bold) and minor task categories:

	**Task**	**Description**	**Further comments**
**Major category**	**Computer – find**		
1.	Client file	Finding client file	Booking visit, ANC follow-up visit, previous pregnancy table<br>Finding the client's file in the eRegistry by running the search function
2.	Lab/ultrasound results	Looking for lab/ultrasound results	Finding lab and/or ultrasound results

Major category	Paper – find		
3.	Client file	Looking for client file	Booking visit, ANC follow-up visit, previous pregnancy table Finding the client's file in archive/storage.
4.	Lab/ultrasound results	Looking for lab/ultrasound results	Lab and/or ultrasound result
**Major category**	**Talking**		
5.	Education & counselling	Only for the pregnant woman	Process of pregnancy and its complications, danger signs in pregnancy, diet and nutrition, rest, exercise in pregnancy, personal hygiene, use of drugs or supplements in pregnancy (e.g. iron and folic acid), care of breasts and breast-feeding, symptoms/signs of labour, plans of delivery, plans for postpartum care, family planning, harmful habits (e.g. smoking, cultural habits), explaining referral procedure
6.	Talking to family	Talking to client's family in the clinic	This may take place both during and/or after consultation hours.
7.	History taking	Demographic information and client history (past medical/surgical, obstetric, family; current pregnancy)	Only report as history-taking if care provider is clearly **not doing anything else than asking/listening** to the client, meaning not writing
8.	Test results from lab/ultrasound	Calling for scheduling tests or results, e.g. lab or ultrasound results from another lab/clinic.	
9.	Clinical support	Talking to colleague about client-related matters, seeking client-related support	Talking to doctor/other nurse about the client, schedule tests (incl. on the phone), meaning not writing
10.	Call client/family	Care provider talks with client or her family on the phone: schedule appointment, getting/conveying results	This may take place both during and/or after consultation hours.
11.	Referrals	Talking related to arranging referrals, high-risk	

		clinic/hospital to notify about referrals	
12.	Technical support	Seeking help in case of technical problems	Technical problems related to either the eRegistry or the internet. Talking to the MCH supervisors/field support
13.	Other		
**Major category**	**Procedures**		
14.	Clinical and/or medical examination	Performing examination	Blood pressure, fundal height, height, weight, pallor, pulse, oedema, breast, temperature, foetal presentation and engagement, foetal heart sound + others. Some of these might take place in another room.
15.	Injections/blood take	Giving injections and taking blood	Most often, tetanus toxoid
16.	Giving tablets		e.g. iron tablets
17.	Other		
**Major category**	**Outside**		
18.	Assisting doctor	Leaving consultation room to go to the doctor's office	When care provider follows the client to the doctor's room (especially in the case of a male doctor), or if the nurse assists the doctor in another room than the consultation room.
19.	Examination in other room	Leaving room to perform examination in another room than the consultation room	
20.	Other	Leaving room to wash hands between consultations	
**Major category**	**Computer – writing**		
21.	Client file (including history)	Entering data into the client's file, including writing during history-taking.	Entering data (from registration, history-taking, examination, lab results) and other documentation in client file, incl. back-up file in case of Internet problems
22.	Lab/ultrasound form	Enter lab/ultrasound results into the system	From lab/ultrasound results paper
23.	Schedule appointment	Write new appointment in the system	Recognises activity either by looking or based on what the care provider is saying
24.	Text message in eRegistry	Writing other places than in the client file, in the eRegistry	E.g. notes, messages to other care providers

**Major category**	**Paper – writing**		
25.	MCH handbook (including history)	Write information in the woman's MCH handbook, including writing during history-taking.	
26.	Client file (history)	Write data, including writing during history-taking.	Write data from history-taking, examination, lab results and other documentation in client file
27.	Register book	Write in the register book	
28.	MCH Handbook/register book	Writing in the MCH handbook at the same time as writing in the register book	If the nurse writes in different places interchangeably
29.	Register book/client file	Writing in the register book at the same time as writing in the client file	If the nurse writes in different places interchangeably
30.	Client file/MCH handbook	Writing in the client file at the same time as writing in the MCH handbook	If the nurse writes in different places interchangeably
31.	Lab/ultrasound/prescriptions/referrals	Write orders	Write orders: lab form, ultrasound, referrals, prescriptions
32.	Schedule next appointment	Write next appointment in the appointment book (schedule book)	
33.	Writing on other paper	Any other writing	
**Major category**	**Computer – read**		
34.	Appointment list	Read client appointments from the system	Read list of appointments in the eRegistry.
35.	Client file	Reading from the client file on the computer	Only reading without typing or writing.
36.	Lab/ultrasound results	Reading lab and/or ultrasound results from computer	Only reading without typing or writing.
37.	Guidelines, treatment	Searching for guidelines, etc. on the computer	Internet search not in the eRegistry platform
38.	Other info	Any other patient- or health information-related reading on the computer	
**Major category**	**Paper – read**		
39.	Appointment list	Read client appointments from appointment book	Read list of appointments in the appointment book.

40.	MCH handbook		
41.	Client file	Reading client information from paper file	Only reading without writing.
42.	Lab/ultrasound results	Reading lab and/or ultrasound results from forms	Only reading without writing.
43.	Guidelines, treatment, official letter	E.g. guidelines, books, journals, official letters	Using books or other literature for guidelines
44.	Other	Any other patient- or health information-related reading on book/paper	
**Major category**	**Between/after consultations**		
45.	Statistics book	Filling in information in the daily statistics book	
46.	Group education		
47.	Cleaning, arranging files	Organising cleaning of equipment, prepare for next client	
48.	Phone/computer: personal	Use of phone/computer for social media, email, etc.	
49.	Other: Eating, praying, toilet etc.	Praying etc.	

How Microsoft Access' data entry form works:
The observer initiates the observation by clicking any of the minor task descriptions under the bold major tasks on the entry form (Figure 1). The click will make the tool start recording the time. The observer then determines the nature of the current activity and clicks the corresponding button on the form followed by the "Confirm entry" button to store the activity. If the observer realises that she misinterpreted an activity and hit the wrong task button, the observer can switch to the correct task button, since the entry of the task is not stored until the "Confirm entry" button is clicked. Similarly, as soon as the care provider switches to a different task or activity, the observer clicks the "Confirm entry" button to complete the current entry. To finish the observation, the observer clicks the "CLOSE" button.

An example may be helpful:
1. Provider starts writing → observer clicks: "computer – writing – file"
2. Provider starts talking (history-taking) → observer clicks: "Confirm entry" → "talking – history-taking"
3. Provider starts writing → observer clicks: "Confirm entry" → "computer – writing – file"

It is important to always click "Confirm entry" before switching the task or before ending the whole observation by clicking "CLOSE".

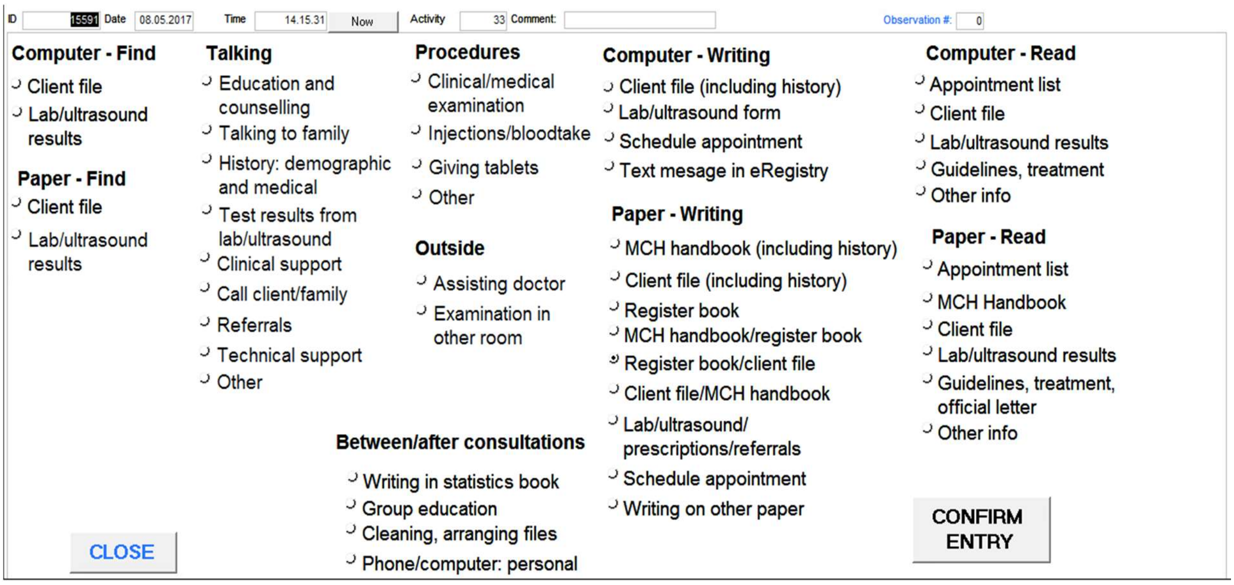

Figure 1: Screenshot of data collection tool

As can be seen both from Table 1 and Figure 1, some of the major categories have an "other" task. This is meant for unexpected activities that the activities described in the tool are unable to capture. For example, if the care provider starts reading something else than any client-related information, then the "Paper – read – other" task button will be pressed. If the observer clicks on an "other" task, then it is important that the observer writes a short comment in the right corner of the tool or on a paper note. This will make the distinction between care- and non-care-related tasks easier to capture.

At the beginning of each observation, the observer will note whether the consultation is a booking or a follow-up visit. For the observations conducted after consultation hours, this should be stored as one single observation.
